# Treatment of Pulpless Teeth

**Published:** 1898-01

**Authors:** A. Schramm

**Affiliations:** Covington, Ky.


### Treatment of Pulpless Teeth.

BY DR. A. SCHRAMM, COVINGTON, KY.

Read before the Cincinnati Academy of Dentistry.

As announced, my subject is the treatment of pulpless teeth by the aid of the compound KOH—sodium and potassium. Allow me to slightly modify this title by saying more concisely, *the treatment of teeth containing putrescent pulps by the aid of KOH.* As is well known, the sodium and potassium method is to-day *only comparatively new*, and while it is, and has been, repeatedly brought before the dental profession of Germany in their journals, there seems to be a lack of interest on part of our American brethren which it certainly merits. It is for this reason that I

again call your attention to it. First—In order to lessen your labors in cleansing putrescent pulp-canals; and, second—because of some experiments and investigations I have been able to make, by courtesy of the Cincinnati College of Dental Surgery in their bacterial laboratory, which I hope will convince you of the indisputable value of this compound. Personally, I have used this compound in daily practice ever since its appearance in America, and can give it my unqualified approval.

As you are probably aware, Dr. Emil Schreyer, of Vienna, was first in recommending sodium and potassium for the sterilization of root-canals. He knew that this compound, when brought into contact with moisture, would rapidly decompose with generation of intense heat. His prime object was to boil the contents of putrescent pulp-canals by the aid of this compound, thereby arriving at sterilization; and he did not recognize the true chemical reaction which took place until the odor of soap apprised him of it.

Root-canal—Treatment as practiced to-day involves a thorough removal of every particle of the putrescent pulp, but in cases where this is impossible, it is certainly desirable and of paramount importance, to find a remedy or a means to render such particles of pulp-debris perfectly aseptic.

Now let us examine our compound in three directions—

First—Does it facilitate the removal of the canal contents?

Second—Does it possess a disinfecting influence upon the canal contents and eventually upon the canal?

Third—Does it successfully remove pulp-debris in those parts of a canal, which can not be reached by ordinary means, and if not, does it leave such pulp-debris in a sterilized condition?

I will answer these questions by the following statements and demonstrations:

When we place into a tooth containing a putrescent pulp a small quantity (Dr. Schreyer says as much as will adhere to the barbs of a broach upon withdrawal from the compound) of sodium and potassium a chemical process occurs, converting the contents into soap and watery glycerine solution. These are easily removed by syringing the canal with water or hydrogen peroxide, using a hypodermic syringe for that purpose. The application of the compound and the syringing is repeated several times. If we now try to enter a canal so treated, we will find ready admission in even such canals as were previously quite impassable to the very finest broach. This is readily accounted for by the fact that the walls of the canal are coated with a soapy film, thereby becoming smooth and slippery. Furthermore, we are cognizant of the fact that this compound is very hygroscopic, and we can therefore feel quite certain that its action extends to the apex of even a sinuous canal. Thus we have converted the putrescent contents of the entire canal into a sterilized innoxious mass. But, in my opinion, sodium and potassium will not only cleanse in this manner the entire canal, but it will enter and immunize by its caustic property the dentinal tubuli, at least a certain distance. And this is one of the cardinal points to which I wish to draw your earnest attention. That sodium and potassium does accomplish this I will presently demonstrate ocularly. But before producing this proof I wish to call your attention to another vital point in results achieved by this method. It is well known that the putrescing pulp and surrounding dentinal tubuli are infested with myriads of bacteria, and we also know that the thermal death point of pathogenic as well as non-pathogenic bacteria is not above 150° C. (315° F.), but that micro-organisms will be effectually destroyed at that temperature. As mentioned in the beginning of this paper, sodium and potassium when brought into contact with moisture decomposes with generation of *intense heat;* and this heat generated in *immediate contact* with bacteria will, in my opinion, certainly destroy the life of these cells.

As to the second point:—Does this compound possess a disinfecting influence upon the canal contents and the canal itself, *i. e.*, the dentinal tubuli? Or, in other words, is it a germicide? This question, and number three, whether it successfully removes all pulp-debris in tortuous canals, and, if not, does it render them sterile, have already been answered in elucidating the effects of sodium and potassium; but to convince you more clearly of its germicidal character, I will give you the ocular demonstrations of experiments made for this purpose:

1. Demonstration of cleansing putrescent pulp-canal with KOH, washing, splitting, mounting.

2. November 24th—About 1 cc. of pure diptheria culture was mixed with equal quantity KOH with a drop of water, and this mixture inoculated upon three sterile tubes of nutrient agar-agar by the stroke cultures. These were placed into incubator for twenty-four hours at 37½° celsius; at the end of that time no growth of organisms.

November 25th—Stroke cultures of mixed mouth bacteria were made on agar-agar in the same manner, about 1 cc. of the bacteria being mixed with an equal quantity of KOH and a drop of water; placed in incubator for twenty-four hours; no growth.

November 26th—Klebs-Loeffler bacillus mixed with same amount of KOH and a drop of water, inoculated to one side of petri-dish containing agar-agar, and a pure culture of diphtheria to other side of same dish. Incubator for twenty-four hours. Sodium, potassium—diptheria mixture; no growth. Pure diphtheria, prodigious growth. Cover glass preparations showing mixed bacteria from mouth, and one from same micro-organisms mixed with KOH.

DISCUSSION.

Dr. Junkerman: In the first place, I want to compliment the essayist on his very estimable and scientific paper. I think his explanations were very concise and definite, and, no doubt, explanations of a very beautiful method of procedure in cases of putrescent pulps. I do not wish to take exception at all, but I would like to ask a question or two, and make what I think, probably a correction or two. I think, in the beginning there was a little misconception or statement that would be liable to mislead.

This preparation he calls a compound; the preparation is not a compound at all; it is a mixture, and it is a mixture of not KOH or NaOH, but it is simply a mixture of K and Na, *i. e.*, potassium and sodium, two well-known metals whose properties are chiefly a great affinity for moisture. In the process, we turn the Na and the K into NaOH and KOH, making what in common terms we call caustic potash and caustic soda mixed, as the essayist says, with other preparations (some form of glycerine I believe), and these substances are formed in a liquid way and very easily washed out of the cavity.

Na and K is not a compound; it is a mixture, and its virtue depends entirely upon the affinity of the two for moisture. I would like to ask the essayist one question, and that is, would not the Na or the K be just as valuable separately as they would be mixed? If not, why does the mixture prove more beneficial than either one of them singly? If either one singly was to be used, I would say that the K was the best to use, because the K has more affinity for water than the Na. But why Dr. Schreyer uses the two together is a question I have never heard answered.

I would take exception, furthermore, to the statement made as regards the infiltration of the dental tubuli; I do not believe he can produce by this process an osmosis, and if he infiltrates this KOH and NaOH, I want to know how he gets that into the dental tubuli. I have sometimes made a statement that I could almost force soft foil, because it was so plastic, into the dental tubuli, but in reality I do not believe we can do that, or force KOH or NaOH into the dental tubuli. The substance in the tubuli is liquid, and might attract the Na and K, but I believe the effect of the K and Na is destroyed before it gets into the tubuli. What I want to know from the essayist is, how he knows that it does get into the dental tubuli.

Dr. Bruchie: The question that Dr. Junkerman asked was a question that was in my mind; I, also, would like to know why the two metals would be more effective than taken separately, inasmuch as either one of the metals has a powerful affinity for moisture. I do not know that I have any further remarks to make, never having experimented with the metals.

Dr. Pfafflin: I would like to ask one question: Why, if this medicament would open up the root-canal as the essayist said it would, so that you could put a broach into it, thereby enlarging the opening—why, when it came to crowding this broach down to the apical foramen with this medicament, would it not open up the apical foramen as well as the more external opening of the root-canal? If the medicament once escaped the root-canal, I would like to know the treatment in such a case as that?

Dr. McLean: Root-canal treatment is quite interesting to me, but there are a few points that the essayist made in his paper that are a little bit difficult to understand. If I remember correctly, he stated that a temperature of over 300° F. occurred when this solution was placed in the canal. I am inclined to believe that the patient would have considerable amusement enjoying life in the dental chair with that temperature in the tooth. I fail to understand how he can control the action of this medicament and prevent it from entering the apical space. If it has such an affinity for the moisture contained therein, I am inclined to believe it would pass through the apical space and create a disturbance the patient would not appreciate. As to the chemical change taking place in contact with moisture, how would it act if there was no moisture? We have sometimes dry canals, and in placing that substance in a dry canal how would it act? When there is a variance in the circumference of the canals the manipulation of the medicament is what I can not understand. Not having had an opportunity of reading the paper prior to hearing it read, it is a little difficult to remember the points of principal interest; but this, particularly, I would like to more thoroughly understand.

Dr. Rauh: This treatment may be very efficacious, and I have no doubt that it is, but if you will try the sulphuric acid treatment of Dr. Callahan, I think you will accomplish the same results; and it now looks to me with possibly less danger of going through the apical space. It seems to me that these substances can not be controlled at all, because, being put in with a broach, it is bound to have its action; and with a tooth such as Dr. Schramm used, I believe he could use almost any method with which I am familiar, and accomplish the same results. I would like to see the thing worked in the numerous cases where we can not find the canals at all, and these are the cases in which we are interested.

Dr. Junkerman: I would explain what seems to be a misapprehension on the part of some. Dr. Schramm did not say that this material widened out the bony part of the root-canal; he simply said that the root-canal was lubricated in such a way by this soapy substance that the broach would slip more easily into it. Sodium and potassium does not decompose tooth tissue. $H_2 SO_4$ does, in reality, dissolve solid tooth tissue; it opens up the canal by eating away the bony tissue; but Dr. Schramm stated that this did not eat the hard tissue of the root-canal, but only the soft tissue, and simply lubricated the canal.

It can readily be seen that Na and K does not open up the apex of the tooth at all; it does not eat the solid tissue of the tooth. Sulphuric acid would be liable to go through the apex because it widens the opening.

Dr. Schramm: Dr. Junkerman has anticipated me in making those remarks. In the first place, I did not say it is a compound; it is called a compound because Dr. Schreyer calls it such, and sells it as such; why he does so I do not know. I am only giving the chemical effects; I do not know why he combines the two. I can not give you chemical analyses of every process that goes on. It is called kalium helicum, the technical name used by most of the foreign dentists who use that remedy; it is also called sodium and potassium, and also called KOH. I am only giving you what I know of the compound and its actions, and what it will accomplish. I said—does it facilitate the opening of a root-canal? It certainly does. Is it a germicide? It certainly is. Does it reach the apical foramen through tortuous canals? It certainly does.

Objections were raised by one or two gentlemen as to the apical space being widened. I never for one moment said you must carry the sodium and potassium to the apex of the root. When this compound is placed in the root a chemical reaction will immediately be set up; I did not say to push it clear to the apex. Moreover, after it has been placed at the entrance to a putrescent pulp-canal, and by its affinity has converted the contents of the dental-canal into soap and watery glycerine solution, you syringe the canal out and do not carry any more sodium and potassium to the apex; you wash out the solution. If the canal is not thoroughly clean, you can introduce some more and then wash. I said distinctly that I made the application several times. I did not make any claim that the canal would be widened. Dr. Callahan's method does affect tooth substance and this does not. This method only makes the canal slippery.

After you have washed the canal with peroxide or water, the broach will enter readily because the walls of the canal are slippery. I did not say you carried any more into it after you have washed it out several times.

You will have on account of the heat (and this answers Dr. McLean's questions) possibly a slight periosteal inflammation, and you will have that with almost any remedy of which I know. You can allay that by the application of cold water however, and the periosteal inflammation will soon subside. The heat of 316° F. does not continue for any great length of time; it is a matter of one, two, three, and it is gone. I can show it to you any day you wish to see an ocular demonstration in the mouth.

As to the other question of Dr. Junkerman, whether it enters the dental tubuli, I say it does enter them. We have here the small fibers which have become decomposed on account of putrescence of the pulp. The dental fibrillæ radiates from the pulp, and to a degree the sodium and potassium will soak into the dental tubuli, and thereby attack the putrescent matter contained within them right around the canal.

I have under the microscope a canal with which I have taken no trouble, and which looks almost as chemically clean as any canal can possibly look. I have taken no particular pains with the demonstration at all.

As regards the apical space, there is no danger. Of course you must not infer that you can handle this in a careless sort of a manner; dentists are not careless people as a rule, and they should not be. You take just as much of the preparation as the barbs on your broach will carry, according to Dr. Schreyer's directions, and thereby you will accomplish just what I have said. That you may enter a tortuous canal with your nerve broach, I did not say, but this remedy will go there.

I can only give you these points, because I have selected this subject, on account of having used it ever since this remedy has been used in America, which has been six or seven years, as it was discussed in the Dental Congress in Chicago in 1893. I have tried Dr. Callahan's method, but I find more inflammation resulting therefrom than I ever have from this, and I feel perfectly confident that you can more readily gain access to your canal and clean it more thoroughly in a shorter time by this method than in any way I know of. If you bring in something better, Dr. Schramm will be the first to adopt it.

